# Prediction of lung tumor types based on protein attributes by machine learning algorithms

**DOI:** 10.1186/2193-1801-2-238

**Published:** 2013-05-24

**Authors:** Faezeh Hosseinzadeh, Amir Hossein KayvanJoo, Mansuor Ebrahimi, Bahram Goliaei

**Affiliations:** Laboratory of biophysics and molecular biology, Institute of Biophysics and Biochemistry (IBB), University of Tehran, Tehran, Iran; Department of Biology at Basic science School & Bioinformatics Research Group, Green Research Center, University of Qom, Qom, Iran

**Keywords:** Lung cancer, Prediction, Structural and physicochemical features, Attributes weighting, Support vector machine, Artificial neural network, Naïve bayes

## Abstract

Early diagnosis of lung cancers and distinction between the tumor types (Small Cell Lung Cancer (SCLC) and Non-Small Cell Lung Cancer (NSCLC) are very important to increase the survival rate of patients. Herein, we propose a diagnostic system based on sequence-derived structural and physicochemical attributes of proteins that involved in both types of tumors via feature extraction, feature selection and prediction models. 1497 proteins attributes computed and important features selected by 12 attribute weighting models and finally machine learning models consist of seven *SVM* models, three *ANN* models and two *NB* models applied on original database and newly created ones from attribute weighting models; models accuracies calculated through *10-fold cross* and *wrapper validation* (just for *SVM* algorithms). In line with our previous findings, dipeptide composition, autocorrelation and distribution descriptor were the most important protein features selected by bioinformatics tools. The algorithms performances in lung cancer tumor type prediction increased when they applied on datasets created by attribute weighting models rather than original dataset. *Wrapper-Validation* performed better than *X-Validation;* the best cancer type prediction resulted from *SVM* and *SVM Linear* models (82%)*.* The best accuracy of *ANN* gained when *Neural Net* model applied on *SVM* dataset (88%). This is the first report suggesting that the combination of protein features and attribute weighting models with machine learning algorithms can be effectively used to predict the type of lung cancer tumors (SCLC and NSCLC).

**Electronic supplementary material:**

The online version of this article (doi:10.1186/2193-1801-2-238) contains supplementary material, which is available to authorized users.

## Introduction

Lung cancer, as a leading cause of death worldwide, starts from the lungs and may spreads to other organs of the body and has a low survival rate of just 15% (Ganesan et al. [Bibr CR72][Bibr CR73] Nomori 2011). It is estimated that at least 1.2 million people were infected with the disease, equivalent to the 12.3% of total number of cancer diagnosed annually, with a mortality rate of 1.1 million people per year (Parkin 2001 Webb-Robertson et al. 2010). As the treatments for each type of lung cancers are different (Motohiro et al. 2002), so if a patient is correctly diagnosed in early stage, the chance for one to be cured will increase (Zhou et al. 2002). The cancer’s types have already been divided into two groups based on pathological and morphological observations: non-small cell lung cancer (NSCLC) (80.4%) and small cell lung cancer (16.8%) (Travis et al. 1995). The diagnosis of tumor type is performed by histology, immunohistochemistry or pathology based on either cells morphology or protein expression. In addition, the underlying genetic aberrations or biological processes may contribute to the cancer malignancy process which cannot be revealed by histological appearance of a tumor (Khan et al. 2001). In order to improve the survival rate, need for an early type detection method of lung tumors have been raised (Delarue and Starr 1967), and this research carried to address this need based on data mining tools.

So far many different techniques such as Chest Radiograph (x-ray), Computed Tomography (CT), Magnetic Resonance Imaging (MRI) and Sputum Cytology have been used for lung cancer classification (Grondin and Liptay 2002 Schaefer-Prokop and Prokop 2002). However, most of these techniques are either expensive and time consuming or applicable only in the advanced stages, when the survival rate of patients is very limited (Fatma et al. 2012). During the last decades, computer-aided cancer classification systems along with the rapid developments of image processing and pattern recognition techniques have been proposed as suitable tools. On the other hand, many researches have looked into the bioinformatics models and data mining algorithms as alternative choices. To improve the accuracy and the speed of lung cancer diagnosis based on radiology, an artificial neural network via hybrid lung cancer detection system named HLND designed (Chiou YSP and Ligomenides 1993). In the other study, a system based on a parameterized two-level convolution artificial neural network have been developed to do this important task (Lin et al. 1996), an automatic method based on the subtraction between two serial mass chest radiographs, which was used in the detection of new lung nodules designed by Hayashibe *et al.* (Hayashibe et al. 1996). Abe *et al.* evaluated another computer-aided diagnosis (CAD) system with automatic detection of pulmonary nodules for lung cancer screening with computed tomography (CT) (Abe et al. 2005). Penedo and *et al.* employed two artificial neural network, one for detecting suspicious regions in a low-resolution image and the other for dealing with the curvature peaks of the suspicious regions, which was used in the detection of lung nodules (Penedo et al. 1998). In the diagnostic systems of lung cancer with computer-aided, the rate of false negative identification should be kept as low as possible to improve the rate of overall identification on the highest possible rate (Zhou et al. 2002).

Machine learning is an automatic and intelligent learning technique that employs variety of statistical tools to “learn” from past data and then use the prior training to classify new data, identify new patterns or predict novel trends (Mitchell 1997). These techniques have been widely used to solve many real world and complex problems (Kerhet et al. 2010). Since their introduction to the bioinformatics community, machine learning approaches helped to accelerate several major researches such as bimolecular structure prediction, gene finding, genomics and proteomics (Zycinski et al. 2011). As these techniques are efficient and inexpensive in solving bioinformatics problems, the applications of them in bioinformatics are becoming popular and continuing to develop (Liu 2004). In recent years machine learning methods have been widely used in prediction especially in medical diagnosis and interestingly. Almost all of these algorithms used in cancer prediction and prognosis employed supervised learning. Furthermore, most of these supervised learning algorithms belonged to a specific category of classifiers that classify on the basis of conditional probabilities or conditional decisions (Cruz and Wishart 2006 Ganesan et al. [Bibr CR72][Bibr CR73]).

One of the most instrumental type of machine learning techniques are *Support Vector Machines* (*SVM* ) which were introduced by Vladimir Vapnik and his colleagues (Bazzani et al. 2001 Baumes et al. 2006). *SVM* s are used for binary classification to find a hyper plane which separates the d-dimensional data perfectly into its two classes (Parsaei and Stashuk 2012 Boswell 2002). In contrast to other classifiers, *SVM* searches for the hyper plane that maximizes the distance from the hyper plane to the nearest examples in each class. An attractive feature of *SVM* is that it can map linearly inseparable data into higher dimensional space where they can be linearly separated. This work is executable with introduction of “kernel induced feature space” notion. Recently, *SVM* has gained much attention as a useful tool for image recognition (Guan et al. 2009 Avci 2012). The use of *SVM*, like any other machine learning technique, involves two basic steps namely training and testing. The first step involves feeding known data to the *SVM* along with previously known decision. It is from the training set that an *SVM* gets its intelligence to classify unknown data (Van Belle et al. 2011). Several studies have already used the performance of *Bayesian* classifier; *artificial neural net* and *SVM* for differentiating obstructive lung diseases, and *SVM* gained the best performance for classification (Lee et al. 2009). It has been shown that *SVM* provide better overall quantification for interstitial lung disease differentiation in high-resolution computerized tomography images (Lim et al. 2011).

The *Naive Bayes (NB)* classifier technique is based on the so-called *Bayesian* theorem and is particularly suited when the dimensionality of the inputs is high. A *Naive Bayes* classifier considers that all attributes (features) independently contribute to the probability of a certain class. This classifier can be trained so efficiently in a supervised learning method and works much better in many complex real-world situations, especially in the computer-aided diagnosis (Gorunescu 2006 Belciug 2008 Dumitru 2009). Despite its simplicity, *Naive Bayes* can often outperform more sophisticated classification methods. The *Bayesian* approach allows scientists to combine new data with their existing knowledge or expertise. Using a training dataset, the *Bayesian* classifiers determine the probability of associating certain classes at certain instances given the values of the predictor variables. *Naive Bayes* classifier provides performances equivalent to other machine learning techniques with low computational effort and high speed (Dumitru 2009).

Herein, regarding the importance of early classification and prediction of lung tumor types in successful treatment of this disease, several machine learning algorithms employed to predict the type of lung cancers based on structural and physicochemical attributes of proteins. Some studies have used sequence-derived structural and physicochemical descriptors in machine learning prediction of structural and functional classes (Dubchak et al. 1999 Karchin et al. 2002 Cai et al. 2003 Cai et al. 2004 Han et al. [Bibr CR49][Bibr CR50]), protein-protein interactions (Bock and Gough 2001 Bock and Gough 2003 Lo et al. 2005 Chou and Cai 2006), subcellular locations (Chou 2000 Chou and Cai 2004 Chou and Shen 2006 Guo and Lin 2006), peptides containing specific properties (Schneider and Wrede 1994 Cui et al. 2007), microarray data (Brown et al. 2000) and protein secondary structure prediction (Ward et al. 2003). Ong and *et al.* showed that currently used descriptors are generally useful for classifying proteins and the prediction performance may be enhanced by combinations of descriptors (Ong et al. 2007); in this experiment, the same datasets as previously reported was used (Hosseinzadeh et al. 2012). Previously feature selection, tree induction and clustering models had been used to classify lung tumors based on important protein features. Follow up of previse work, the application of three machine learning models practiced here to introduce accurate prediction tools for lung cancer types based on important attributes of related proteins.

## Results

### Data preparation and feature selection

Proteins that involved in two types of lung tumors obtained from conversion of gene symbols defined by microarray analysis in the GSEA db, using DAVID server. The list of genes associated with two types of lung tumors and those that were common between them showed in Table 1.

**Table 1:** **The list of overexpressed genes in three classes of lung tumors (SCLC, NSCLC and COMMON) defined by microarray analysis; extracted from GSEA db**

Tumor type	Gene Symbol
SCLC	APAF1, BCL2, BCL2L1, BIRC2, BIRC3, CCNE1, CCNE2, CDK2, CDKN1B, CDKN2B, CHUK, CKS1B, COL4A1, COL4A2, COL4A4, COL4A6, CYCS, FN1, IKBKB, IKBKG, ITGA2, ITGA2B, ITGA3, ITGA6, ITGAV, ITGB1, LAMA1, LAMA2, LAMA3, LAMA4, LAMA5, LAMB1, LAMB2, LAMB3, LAMB4, LAMC1, LAMC2, LAMC3, MAX, MYC, NFKB1, NFKBIA, NOS2, PIAS1, PIAS2, PIAS3, PIAS4, PTEN, PTGS2, PTK2, RELA, SKP2, TRAF1, TRAF2, TRAF3, TRAF4, TRAF5, TRAF6, XIAP
NSCLC	ARAF, BAD, BRAF, CDKN2A, EGF, EGFR, ERBB2, FOXO3, GRB2, HRAS, KRAS, MAP2K1, MAP2K2, MAPK1, MAPK3, NRAS, PDPK1, PLCG1, PLCG2, PRKCA, PRKCB, PRKCG, RAF1, RASSF1, RASSF5, SOS1, SOS2, STK4, TGFA
COMMON	AKT1, AKT2, AKT3, CASP9, CCND1, CDK4, CDK6, E2F1, E2F2, E2F3, FHIT, PIK3CA, PIK3CB, PIK3CD, PIK3CG, PIK3R1, PIK3R2, PIK3R3, PIK3R5, RARB, RB1, RXRA, RXRB, RXRG, TP53

#### Data cleaning

In original dataset, 59 records classified as SCLC, 30 records belonged to NSCLC class and 25 other records to COMMON tumor classes. For each record 1497 features computed and after removing duplicate, useless and correlated attributes, the number of protein features for each record decreased to 1089 features (less than 28% removed) and this cleaned dataset named as *Final Cleaned database* (*FCdb*).

#### Feature selection

**Figure 1:**
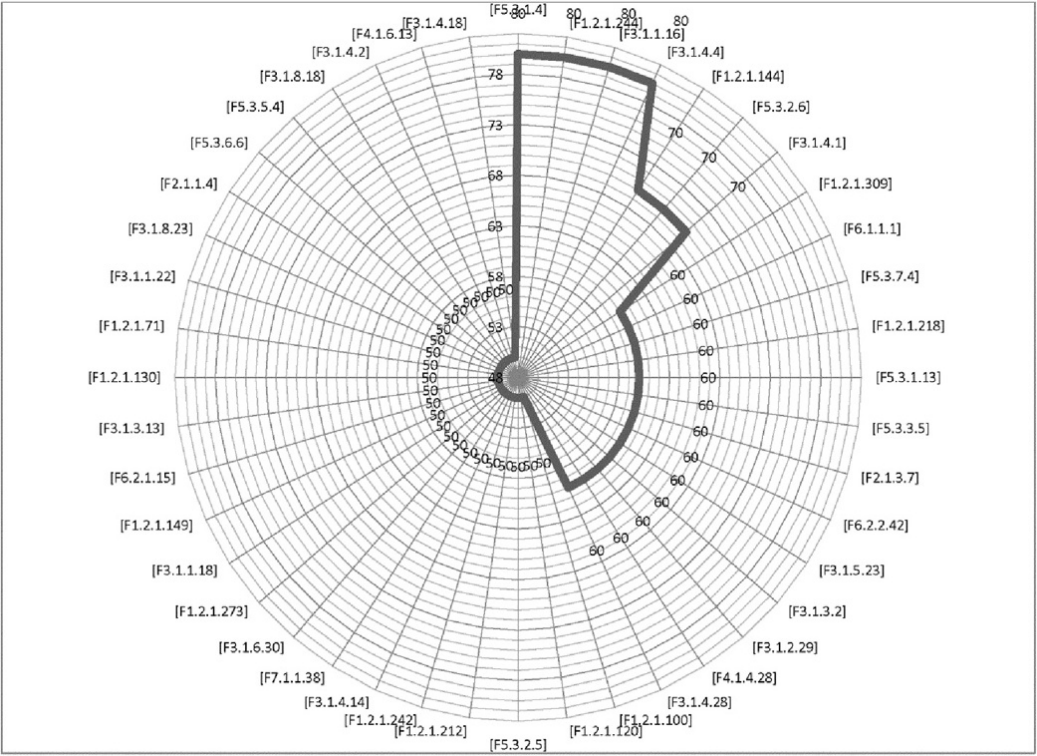
**The most important protein attributes selected by more than fifty percent of attribute weighting algorithms.** As is evident, the features of distribution descriptor (F5.3), dipeptide composition (F1.2) and autocorrelation (F3.1) were defined important by 80% of attribute weighting models.

**Figure 2:**
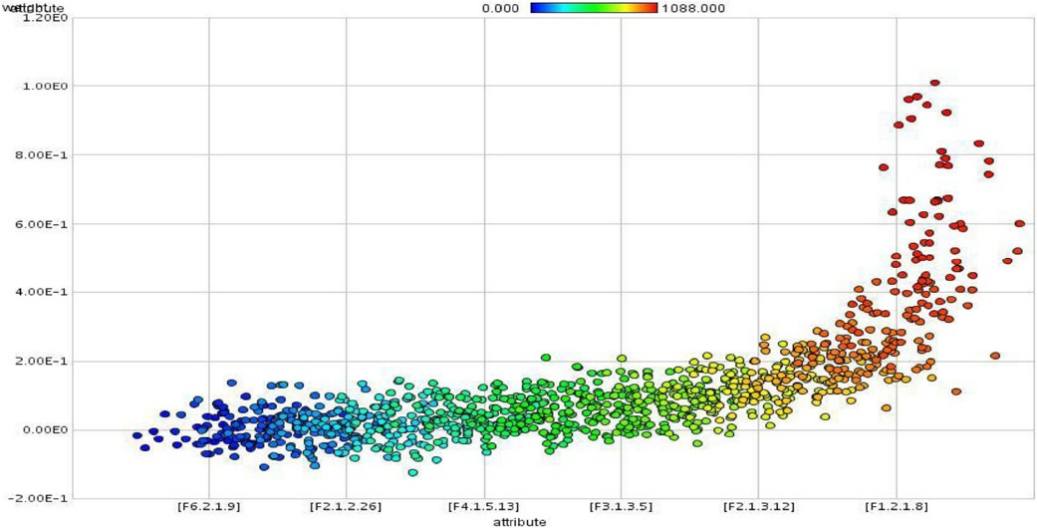
**Dispersion of protein attributes that gained weight value between 0 to 1 by attribute weighting model of SAM (the index of protein attributes exactly defined in Additional file** **1** **: Table S1).**

**Figure 3:**
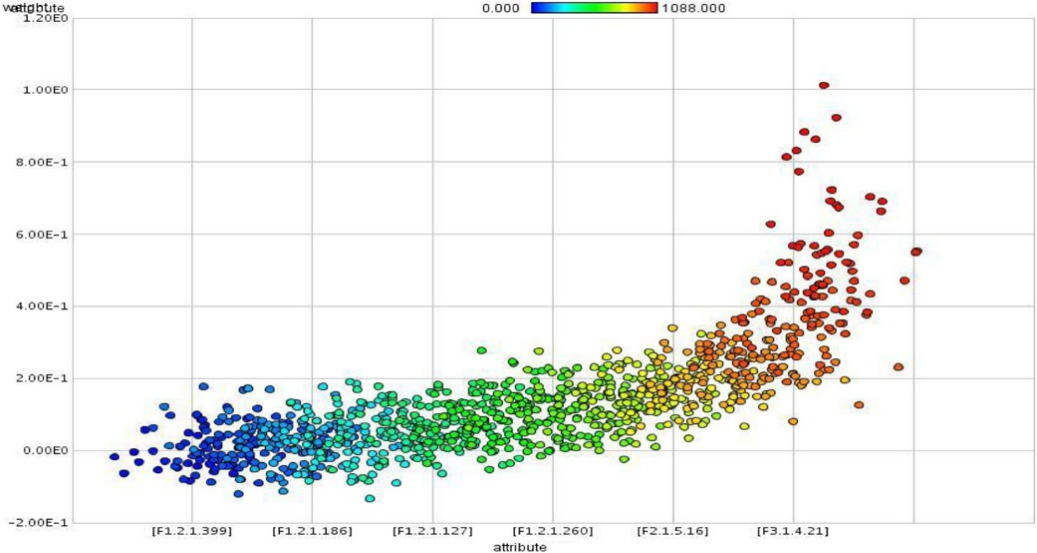
**Dispersion of protein attributes that gained weight value between 0 to 1 by attribute weighting model of Maximum Relevance (the index of protein attributes exactly defined in Additional file** **1** **: Table S1).**

Twelve attributes weighting models applied on *FCdb* which gave each feature a weight between 0 to 1. Features that gained weight values higher than 0.50 with at least 50% of weighting algorithms regarded as important protein features. Figure 1 showed the most important protein attributes selected by more than 50 percent of attribute weighting algorithms (*Information gain, Information gain ratio, Rule, Deviation, Chi Squared, Gini index, Uncertainty, Relief, SVM and PCA*). Dispersions of features’ weight values by two other weighting models (*SAM and Maximum Relevance*) have illustrated in the Figure 2 and Figure 3.

### Classification and prediction

#### Support vector machine approach

Gained accuracies and *Kappa* values for each *SVM* model (while Gamma and C set as 0.0065 and 10, respectively and ran with X-validation approach) on 13 datasets (*FCdb* and 12 datasets that obtained from attribute weighting application: *Information gain, Information gain ratio, Rule, Deviation, Chi Squared, Gini index, Uncertainty, Relief, SVM, PCA, SAM and MR*) illustrated in the Table 2. Furthermore, Table 3 shows the results of running seven *SVM* and *wrapper validation* methods on datasets that derived from attribute weighting (this model cannot be applied on main dataset, *FCdb*, as required attribute weighted datasets). When *X-validation* (ten-fold cross validation) applied, the average accuracy ranged from 32.27% (*SVM Hyper*) to 67.36% (for *SVM* and *SVM Linear*), while the lowest and highest accuracies accounted for the same algorithms (30.0% and 81.67%, respectively). The *Kappa* index was followed the same pattern, the lowest came from *SVM Hyper* (-6.10%) and the highest from *SVM* and *SVM Liner* (69.09%). With *Wrapper validation* application, the average accuracies ranged from 33.21% (for *SVM Hyper*) to 69.53% (*SVM)* and the minimum and maximum accuracies (23.86% and 71.97%) were again for the same models, respectively (Figure 5).

**Table 2:** **The total accuracy and Kappa obtained from applying seven** ***SVM*** **algorithms with** ***X-validation*** **on 13 datasets (FCdb and 12 datasets that obtained from attribute weighting models)**

***SVM*** models		***SVM***	***SVM*** Linear	***SVM*** Evolutionary	***SVM*** Lib ***SVM***	***SVM*** PSO	***SVM*** Fast Large Margin	***SVM*** Hyper
Datasets								
FCdb	Accuracy	67.42%	67.42%	47.12%	51.67%	45.30%	64.77%	33.26%
	Kappa	40.85%	40.85%	25.76%	0.00%	0.00%	44.54%	2.48%
Chi Squared	Accuracy	70.30%	70.30%	55.91%	61.36%	47.50%	68.48%	32.42%
	Kappa	44.09%	44.09%	29.36%	31.01%	18.46%	46.71%	2.21%
Deviation	Accuracy	50.68%	50.68%	58.71%	51.97%	49.24%	34.02%	31.44%
	Kappa	2.08%	2.08%	32.56%	13.00%	22.16%	0.06%	-0.24%
Gini Index	Accuracy	67.42%	67.42%	64.77%	63.94%	43.86%	63.18%	31.44%
	Kappa	42.19%	42.19%	45.05%	31.19%	0.00%	42.74%	-0.24%
Info Gain	Accuracy	74.39%	74.39%	60.30%	65.61%	45.30%	63.18%	31.44%
	Kappa	54.43%	54.43%	38.77%	34.02%	0.00%	43.16%	-0.24%
Info Gain Ratio	Accuracy	65.76%	65.76%	64.62%	54.17%	45.30%	67.42%	34.17%
	Kappa	34.46%	34.46%	45.06%	6.46%	0.00%	47.55%	-1.65%
PCA	Accuracy	50.68%	50.68%	58.71%	51.97%	49.24%	34.02%	31.44%
	Kappa	2.08%	2.08%	32.56%	13.00%	22.16%	0.06%	-0.24%
Relief	Accuracy	71.29%	71.29%	58.03%	56.89%	56.36%	73.79%	30.00%
	Kappa	47.88%	47.88%	26.55%	13.61%	26.89%	56.22%	-1.21%
*SVM*	Accuracy	81.67%	81.67%	59.55%	66.74%	43.79%	78.18%	36.14%
	Kappa	69.09%	69.09%	34.98%	40.22%	0.00%	64.73%	3.19%
Uncertainty	Accuracy	69.32%	69.32%	61.14%	58.64%	45.30%	64.55%	32.35%
	Kappa	44.57%	44.57%	39.90%	16.82%	0.00%	43.04%	1.22%
Rule	Accuracy	64.92%	64.92%	59.39%	51.67%	45.30%	61.14%	31.36%
	Kappa	36.01%	36.01%	37.90%	0.00%	0.00%	38.09%	-6.10%
SAM	Accuracy	62.20%	62.20%	56.67%	52.50%	46.21%	54.77%	33.26%
	Kappa	31.04%	31.04%	36.30%	2.00%	0.00%	26.61%	2.48%
MR	Accuracy	78.03%	78.03%	58.86%	63.03%	53.64%	76.36%	32.65%
	Kappa	63.43%	63.43%	28.59%	31.73%	24.33%	61.02%	-4.27%

**Table 3:** **The total accuracy obtained from running seven** ***SVM*** **methods and** ***X-wrapper validation*** **on the 12 datasets derived from attribute weighting models**

***SVM*** models	***SVM***	***SVM*** Linear	***SVM*** Lib ***SVM***	***SVM*** Evolutionary	***SVM*** POS	***SVM*** Hyper	***SVM*** Fast Large Margin
Dataset							
SAM	70.98%	68.86%	51.67%	41.97%	45.68%	41.82%	66.52%
MR	70.38%	70.00%	51.67%	47.35%	40.15%	35.15%	69.32%
Chi Squared	69.02%	70.83%	51.67%	43.79%	44.39%	32.58%	71.89%
Deviation	67.80%	71.14%	51.67%	47.42%	39.24%	40.38%	67.12%
Gini Index	63.94%	68.18%	51.67%	45.76%	47.12%	32.73%	68.56%
Info Gain	70.00%	70.98%	51.67%	45.83%	49.17%	42.95%	70.30%
Info Gain Ratio	71.97%	68.48%	51.67%	46.59%	43.79%	23.86%	67.50%
PCA	70.15%	68.26%	51.67%	45.68%	41.29%	29.77%	72.73%
Relief	70.83%	66.74%	51.67%	45.83%	37.95%	28.26%	70.23%
Rule	71.89%	68.33%	51.67%	47.58%	43.71%	28.03%	72.88%
*SVM*	70.00%	68.26%	51.67%	47.35%	44.02%	33.03%	67.42%
Uncertainty	67.42%	69.17%	51.67%	47.35%	43.48%	29.92%	66.44%

#### Artificial neural network

The results of running three models of *ANN* (*Auto MLp*, *Neural Net* and *Perceptron*) on 13 datasets given in the Table 4. The most accurate model was *Neural Net* when applied on *SVM* dataset with accuracy of 87.73%. The ranges of accuracies in three models of artificial neural network were respectively 52–86, 53–83 and 31 – 59 percents. The percentage of *Kappa* in the *Auto MLP* model except for *PCA* and *Deviation* datasets started from 46% to went up to 77%. The maximum and minimum percent of *Kappa* in the *Neural Net* model (except for *PCA* and *Deviation* datasets) were respectively 80% and 43%. *Kappa* index in *Perceptron* model was lower than two other models and its maximum got to to 26%.

**Table 4:** **The total accuracy and Kappa index obtained from three Neural Network models on 13 datasets (FCdb and 12 datasets that obtained from attribute weighting models)**

Data Base	Auto MLp Accuracy	Neural Net Accuracy	Perceptron Accuracy	Auto MLp Kappa	Neural Net Kappa	Perceptron Kappa
Chi Squared	73.79%	70.23%	54.09%	56.39%	51.54%	24.84%
Info Gain Ratio	80.76%	83.41%	52.58%	68.53%	71.55%	13.60%
FCdb	69.24%	81.59%	50.76%	50.05%	69.36%	3.43%
*SVM*	85.15%	87.73%	57.80%	75.66%	79.66%	20.61%
Uncertainty	82.58%	81.59%	52.42%	71.33%	69.92%	18.58%
PCA	51.67%	51.67%	30.98%	0.77%	0.00%	-5.04%
Relief	77.27%	75.61%	51.67%	62.52%	60.30%	16.09%
Rule	76.06%	80.53%	48.03%	60.96%	67.58%	5.45%
Deviation	51.67%	52.50%	30.98%	1.73%	3.33%	-5.04%
Gini Index	76.29%	76.21%	48.86%	61.62%	61.97%	11.38%
Info Gain	85.91%	85.98%	51.44%	76.98%	77.09%	16.98%
SAM	66.32%	64.62%	52.65%	45.56%	42.92%	15.51%
MR	76.29%	75.45%	58.56%	61.88%	60.12%	25.92%

#### Naïve bayes

As shown in the Table 5, the performance of *NB* models was lower when compared to *SVM* and *ANN* algorithms. The best accuracy gained with *NB* models was 77% and belonged to *Naïve Bayes* model ran on *SVM* and *Maximum Relevance* datasets. The *Bayes Kernel* model performance on 13 datasets differ from 54% to 70% and the same ranged from 44% to 77% when *Naïve Bayes* model applied (except for *FCdb*). The *Kappa* index again was lower than accuracy and its maximum and minimum values were 63% and 11%.

**Table 5:** **The total accuracy and Kappa index obtained from two Naïve Bayes models on 13 datasets (FCdb and 12 datasets that obtained from attribute weighting models)**

Data Base	Bayes Kernel Accuracy	***Naive Bayes*** Accuracy	Bayes Kernel Kappa	***Naive Bayes*** Kappa
Rule	57.93%	48.03%	13.54%	25.30%
*SVM*	66.97%	77.35%	42.20%	63.30%
Uncertainty	58.21%	55.45%	14.33%	32.65%
Relief	66.74%	72.65%	42.94%	55.55%
PCA	54.39%	44.02%	19.52%	11.38%
Info Gain Ratio	61.24%	61.14%	25.22%	41.00%
Info Gain	69.32%	70.23%	44.63%	54.66%
Gini Index	65.68%	66.74%	38.20%	48.97%
Deviation	54.39%	44.02%	19.52%	11.38%
Chi Squared	63.18%	64.09%	37.20%	42.02%
FCdb	58.21%	14.30%	42.20%	32.60%
MR	70.00%	77.20%	50.84%	63.60%
SAM	61.20%	62.95%	26.95%	39.75%

## Discussion

Lung cancer is considered as the main cause of cancer death worldwide, and detection of this disease in its early stages is difficult because symptoms appear only at advanced stages causing the mortality rate to be high(Fatma et al. 2012). The 5-year survival rate of localized stage, when the cancer does not spread to additional sites like lymph nodes or other parts of body, is about 50%. Various factors influencing 5-year survival rate such as the stage of cancer, the type of cancer, general health, etc. Early detection of lung cancer is the leading factor decreasing mortality rate and increasing in survival rate (Fontana et al. 1986). Histologically, about 80% of lung cancer are from NSCLC class and just 20% are identified as SCLC cancers (Hu et al. 2002). The pathological distinction between NSCLC and SCLC tumors is so important because patients with NSCLC tumor are treated differently from those with SCLC tumors (Garber et al. 2001). Detection of lung cancer in its early stage is the key in curing patient and automated diagnosis would play crucial roles in this matter (Ganesan et al. [Bibr CR72][Bibr CR73]).

So far many scientists tried to propose new methods to classify the types of lung cancer in early stages (Edwards et al. 2000 Petersen and Petersen 2001 Beadsmoore and Screaton 2003 Boffa 2011 Anagnostou et al. 2012 Gilad et al. 2012 West et al. 2012). In some studies, bioinformatics or data mining models have been used. For example, a training–testing approach has been used to test the reliability of cDNA microarray-based classifications of resected human NSCLCs analyzed (Yamagata et al. 2003). Artificial neural networks have already been widely exploited in computer-aided lung cancer diagnosis, classifing of individual lung cancer cell lines (SCLC and NSCLC) based on DNA methylation markers by using linear discriminant analysis and artificial neural networks (Marchevsky et al. 2004). Neural network have also been used for lung cancer diagnosis to help oncologists to plan for a better medication and early diagnosis (Ganesan et al. [Bibr CR72][Bibr CR73]). The color and texture features from images have also been used as tools for the classification of lung cancer using artificial neural network (Almas and Bariu, 2012). Furthermore, lung cancer gene expression database analysis incorporated prior knowledge with support vector machine-based classification method into cancer classification (Guan et al. 2009). The use of machine learning in cancer detection and prediction is investigated in another study (Lipson et al. 1961). Machine learning techniques like artificial neural network and decision tress are used for cancer detection for nearly 20 years (Galeotti et al. 1986 Campanella 1992 Liu et al. 2006). The potential of using machine learning methods for detecting lung cancer cells or tumors via X-rays, Computed Tomography (CT) has been elaborated in other studies (van Ruth et al. 2003 Kancherla and Mukkamala 2011,2012). They have also been used for tumor classification or cancer detection using microarray data or gene expression are Fisher Linear Discriminant analysis (Brown and Botstein 1999), K Nearest Neighbor (KNN) (Dudoit et al. 2002), (*SVM* ) (Peterson and Ringner 2002), boosting, and self-organizing maps (SOM) (Eisen et al. 1998), hierarchical clustering (Tamayo et al. 1999), and graph theoretic approaches (Sakas et al. 2007).

A significant number of researchers have worked on the ensemble of the multiple classifiers to improve the performance of classification of cancer (Abbass 2002 Zhou et al. 2002 Futschik et al. 2003 Santos-Garcia et al. 2004 Hong-HeeWon 2007). The ensemble classifier increases not only the performance of the classification, but also the confidence of the results. Zhou and *et al.* propose an automatic pathological diagnosis procedure named NED, which utilizes artificial neural network ensemble to identify lung cancer cells in the images of the specimens of needle biopsies (Zhou et al. 2002).

Regarding the importance of distinction between lung cancer tumors and need for finding new simple and effective methods for lung cancer types’ detection, the classification and prediction of lung cancers based on structural and physicochemical descriptors of proteins have been proposed by using machine learning models, as sequence-derived structural and physicochemical descriptors may be highly useful for representing and distinguishing proteins or peptides of different structural irrespective of sequence similarity (Cai et al. 2003 Han et al. [Bibr CR49][Bibr CR50] Lo et al. 2005 Li et al. 2006) too long sentence.

The first step for fulfilling to this idea is identification and selection of most important and appropriate features. PROFEAT is a very trusty server for computing sequence-derived structural and physicochemical descriptors (Rao et al. 2011), so 1497 attributes of SCLC and NSCLC proteins computed. Twelve different attribute weighting models applied to final cleaned dataset; as each algorithm uses a specific pattern to define the most important features, thus, the results may be different (Baumgartner et al. 2010 Ebrahimi et al. 2011 Ebrahimie et al. 2011 Hosseinzadeh et al. 2012). As is shown in Figure 1, inline with our previous published study (Hosseinzadeh et al. 2012), the most important feature groups that selected by 80% of models were from F5.3 (distribution descriptors), F1.2 (dipeptide composition) and F3.1 (autocorrelation) groups. Furthermore, Figure 2 and Figure 3 show that by running two additional weighting models, F1.2 (dipeptide composition), F2.1 (autocorrelation) and F3.1 (autocorrelation) were also selected as important features. These features can be effectively used to distinguish between different types of lung tumors. As is proven in the other study, feature selection reduces the number of features and improves the accuracy (Kancherla et al. 2009), here also the potential use of feature selection to improve the accuracy and efficiency of lung cancer detection is confirmed.

In next step, classification and prediction of lung tumors based on structural and physicochemical properties of associated proteins performed and several prediction models such as *SVM, ANN* and *NB* used. Seven prediction models of support vector machines (*SVM, LibSVM, SVM Linear, SVM Evolutionary, SVM PSO, SVM Fast Large Margin and SVM Hyper Hyper*) applied on 13 datasets (main dataset, *FCdb,* and 12 other datasets that generated from attribute weighting algorithms: *Information gain, Information gain ratio, Rule, Deviation, Chi Squared, Gini index, Uncertainty, Relief, SVM, PCA, SAM* and *Maximum Relevance*). Two validation algorithms, *X-validation* and *X-wrapper,* applied on datasets to calculate the models performance and accuracies (Tables 2 and 3). The findings showed *SVM Hyper* performance was the worst and this model even was less accurate than chance models (average 33.21%). Two other models (*SVM* and *SVM Linear*) showed the best performance and their accuracies reached up to 82%. When the results of two validation methods (*X-Validation* and *Wrapper Validation*) compared, generally the performance of *X-Validation* was better than X-W*rapper Validation,* although the *Wrapper* performed better when applied on *SVM , SVM Linear* and *SVM Fast* models. The best accuracies gained when *X-Validation* applied on dataset created from *SVM* attribute weighting but for *Wrapper-Validation* the datasets were *Deviation, Relief* and *Rule.* The results suggested that either *SVM* or *SVM Linear* would be the best candidate algorithms to predict lung cancer if they apply on *SVM* datasets.

The results of three *ANN* application (*Auto MLp*, *Neural Net* and *Perceptron)* showed *Neural Net* was the best and the most accuracte model when it agained applied on *SVM* dataset, while the worst performance belonged to *Perceptron* model; the accuracies of *Auto MLp* and *Perceptron* models were high and nearly at the same levels (86% and 58%) when they applied on *Information Gain* and *SVM* datasets. Generally the *Kappa* indexes were less accurate, the best index obtained from three models *Auto MLp*, *Neural Net* and *Perceptron* were respectively 77%, 80% and 26%; therefore the best index gained from *Neural Net* model, too.

As shown in Table 5, the best accuracy and *Kappa* index of *Naïve base* and *Naïve base kernel* models gained when they ran on *Maximum Relevance* dataset (77%), and again the indices were lower. The results confirmed that *Naïve base* model was is better than *Naïve base kernel.*

## Conclusions

Comparing the performances of three types of machine learning models (*SVM* , *ANN* and *NB*) to predict and detect the type of lung tumors based on structural and physicochemical attributes of proteins showed that the *Neural Net* model ran on *SVM* dataset gained the best accuracy (88%). Our results showed the potential use of feature selection and prediction models can be effectively used as a simple application. The results also showed that attribute weighting can be beneficiary both to processing time and getting more accurate results.

Dipeptide composition, Moran autocorrelation and distribution descriptor were the most important protein features selected by weighting tools. To our best knowledge, the findings of this study for the first time showed that protein features in combination with machine learning algorithms can be effectively used to determine any types of lung cancer tumors.

## Materials and methods

### Data preparation and feature selection

As shown in our previous study (Hosseinzadeh et al. 2012), the over represented genes in any type of lung tumors obtained from microarray GSEA db (Gene Set Enrichment Analysis database); a well-known database used for storing the results of experimental microarray analysis and determines whether contains a section of Molecular Signatures Database (MSigDB) that is a collection of annotated gene sets for use with GSEA software. It made possible to search for gene sets, examine gene sets and their annotations and download them (Subramanian et al. 2005). A list of appropriate gene lists defined and downloaded (for more details in (Hosseinzadeh et al. 2012). The gene sets converted to protein accession numbers by using DAVID server (http://david.abcc.ncifcrf.gov) and protein sequences extracted from UniProt Knowledgebase (Swiss-Prot and TrEmble) afterwards.

#### Structural and physicochemical attributes

Seven types of of proteins features that were involved in three classes of lung tumors (SCLC, NSCLC and COMMON) were calculated by using PROFEAT web server facilities. These features included of (1) amino acid composition, dipeptide composition. (2) Normalized Moreau–Broto autocorrelation; (3) Moran autocorrelation; (4) Geary autocorrelation; Autocorrelation descriptors are defined from the distribution of amino acid properties along the sequence. The amino acid indices used in these autocorrelation descriptors included hydrophobicity scales, average flexibility indices, polarizability parameter, and free energy of solution accessible surface area in trepeptide, residue volume, steric parameter, and relative mutability. (5) the composition (C), transition (T) and distribution (D) of various structural and physicochemical properties; These descriptors are derived for each of the following physicochemical properties: hydrophibicity, polarity, polarizibility, charge, secondary structures, and normalized Van der Waals volume. (6) sequence- order-coupling number, quasi sequence-order attributes; The quasi-sequence-order descriptors are derived from both the Schneider-Wrede physicochemical distance matrix and the Grantham chemical distance matrix between the 20 amino acids. (7) pseudo amino acid composition; Instead of using the conventional amino acid composition to represent the sample of a protein, Chou proposed the pseudo amino acid (PseAA) composition in order to include the sequence-order information. Therefore one thousands and ninety seven protein features or attributes computed by PROFEAT web server (http://jing.cz3.nus.edu.sg/cgi-501bin/prof/prof.cgi). An index of Fi.j.k.l is used to represent the l^th^ descriptor value of the k^th^ descriptor of the j^th^ feature in the i^th^ feature group, which serves as an easy reference to the PROFEAT manual provided in the server homepage (Li et al. 2006). A complete list of these feature indices and their complete definition is shown in the Addtional file 1: Table S1 (Hosseinzadeh et al. 2012).

#### Running data mining models

A dataset of 1497 features of three groups of protein was imported into Rapid Miner software (Rapid Miner 5.0.001, Rapid-I GmbH, Stochumer Str. 475, 44227 Dortmund, Germany), and the type of tumor was set as target or label attribute.

#### Data cleaning

Duplicate and useless features removed and the new database was labeled as the *Final Cleaned database* (*FCdb*).

#### Attribute weighting

To identify the most important features, 12 attribute weightings algorithms applied to the *FCdb* (they were: *weight by Information gain, weight by Information Gain ratio, weight by Rule, weight by Deviation, weight by Chi squared statistic, weight by Gini index, weight by Uncertainty, weight by Relief, weight by SVM (Support Vector Machine)* and *weight by PCA (Principle Component Analysis)* (for more details see (Hosseinzadeh et al. 2012)). Two more attribute weighting models of *SAM* and *MR* are also applied in this study with the following definition:

**Weight by*Significance analysis of microarrays***:*SAM* is a statistical technique; introduced in 2001; which used to determine whether changes in gene expression are statistically significant or not. With the advent of DNA microarrays it is now possible to measure the expression of thousands of genes in a single hybridization experiment. Generated data is huge and introducing such a model is essential.

**Weight by*Maximum Relevance***: The Max-Dependency feature selection can be efficiently implemented as the Minimum Redundancy and Maximum Relevance (mRMR) algorithm. Significantly outperforms the widely used max-relevance selection method: mRMR features cover a broader feature space with fewer features. mRMR is very efficient and useful for gene selection and many other applications. Both relevance and redundancy estimation are low dimensional problems (i.e. involving only 2 variables). This is much easier than directly estimating multivariate density or mutual information in the high dimensional space, this algorithm is faster speed and more reliable estimation.

#### Attribute selection

After running attribute weighting models on *FCdb*, each protein attribute gained a weight (between 0 and 1) showing its importance with regards to the target attribute (type of tumors). All variables that obtained weight values higher than 0.50 were selected and 12 new datasets created. These newly formed datasets were named according to their attribute weighting models.

### Classification and prediction

In our previous study, after running feature selection, several decision tree induction models and unsupervised clustering algorithms employed to identify the most important protein attributes and obtaining the best classification of lung tumors based of them, but here in this study we used machine learning methods to predict the type of lung tumor based on machine learning and training capabilities.

#### Support vector machine approach

*SVM* s are popular and powerful supervised data classification and prediction techniques with associated learning algorithms which analyze data and recognize patterns. Basic *SVM* takes a set of input data and predicts, for each given input, which of two possible classes forms the output, making it a non-probabilistic binary linear classifier. Given a set of training examples, each marked as belonging to one of two categories, a *SVM* training algorithm builds a model that assigns new examples into one category or the other. Herein we used seven models of *SVM* algorithms (*SVM, LibSVM, SVM Linear, SVM Evolutionary, SVM PSO, SVM Fast Large Margin and SVM Hyper Hyper*) on 13 datasets to predict the type of lung tumors based on sequence-derived structural and physicochemical descriptors of proteins that involved in different types of lung tumors. *LIBSVM* is an integrated software for support vector classification, (C-SVC, nu-SVC), regression (epsilon-SVR, nu-SVR) and distribution estimation (one-class SVM). It supports multi-class classification. *Linear SVM* is the newest extremely fast machine learning (data mining) algorithm for solving multiclass classification problems from ultra large data sets that implements an original proprietary version of a cutting plane algorithm for designing a linear support vector machine. *Linear SVM* is a linearly scalable routine meaning that it creates an *SVM* model in a CPU time which scales linearly with the size of the training data set. *Evolutionary support vector machines (ESVMs)* are novel techniques, these methods incorporate the learning engine of the up to date *SVMs* but develop the coefficients of the decision function by means of evolutionary algorithms (*EAs*) (Stoean, Stoean et al. 2011). *PSO (Particle Swarm Optimization*) algorithms make particles with fitness values which are evaluated by the fitness function to be optimized. PSO is initialized with a group of random particles (solutions) and then searches for most efficient particles by updating each generation (Ardjani and Sadouni 2010). Applies a *fast margin learner based on the linear support vector* learning scheme proposed by R.-E. Fan, K.-W. Chang, C.-J. Hsieh, X.-R. Wang, and C.-J. Lin. Although the result is similar to those delivered by classical *SVM* or logistic regression implementations, this linear classifier is able to work on data set with millions of examples and attributes. It is well-known that *SVM* can be properly used for two-way classification. *Hyper SVM* (Shyu and Liao model solve this problem that how can we decide which parameter order can be changed to reproduce a new classification. This model is included a Huffman-Tree like mechanism, called *hyper SVM*^2011^). Briefly, main database (FCdb) transformed to *SVM* format and scaled by grid search (to avoid attributes in greater numeric ranges dominating those in smaller numeric ranges) and to find the optimal values for operator parameters. Dataset divided into 10 parts and 9 parts used as training set and the last part as testing set.

#### Validation methods

To prevent over-fitting problems, *X-validation* and *X-wrapper validation* methods applied and the procedure repeated for 12 different testing sets (*Information gain, Information gain ratio, Rule, Deviation, Chi Squared, Gini index, Uncertainty, Relief, SVM, PCA, SAM and Maximum Relevance*) and then the average of accuraies and *Kappa* indices computed. The performance evaluator operator used for classification tasks (in cases where the label attribute has a binominal value type) and for polynominal classification tasks. Other polynominal classification tasks such as Polynominal Classification Performance Evaluator (PCPE) operator employed and accuracy and *Kappa* statistics calculated.

#### Kernel trick models

**Figure 4:**
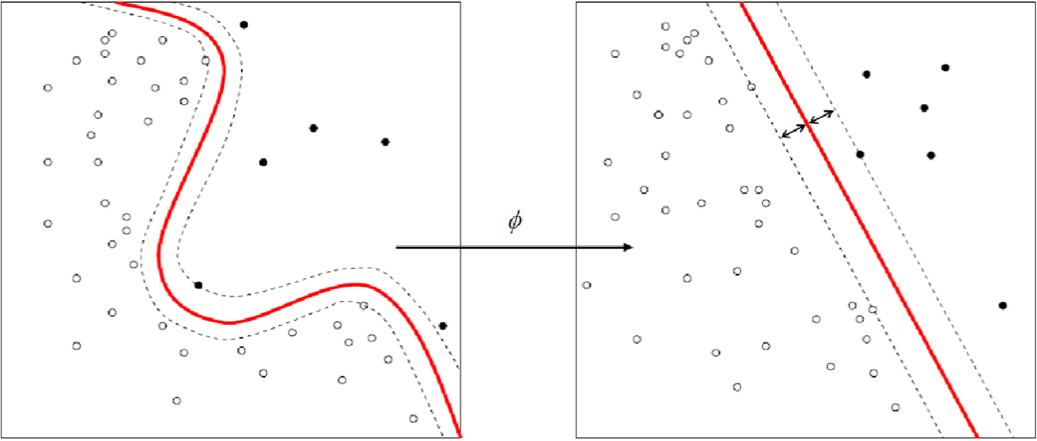
**The mechanism of Kernel trick models.** These machines are used to compute a non-linearly separable function into a higher dimension linearly separable function.

In addition to performing linear classification, *SVM* s can efficiently perform non-linear classification using what is called the kernel trick, implicitly mapping their inputs into high-dimensional feature spaces. The original optimal hyperplane algorithm proposed was a linear classifier. However, later on it was suggested a way to create nonlinear classifiers by applying the kernel trick to maximum-margin hyperplanes. The resulting algorithm is formally similar, except that every dot product is replaced by a nonlinear kernel function. This allows the algorithm to fit the maximum-margin hyperplane in a transformed feature space. For machine learning algorithms, the **kernel trick** is a way of mapping observations from a general set *S* into an inner product space *V* (equipped with its natural norm), without ever having to compute the mapping explicitly, in the hope that the observations will gain meaningful linear structure in *V*. Linear classifications in *V* are equivalent to generic classifications in *S*. The trick to avoid the explicit mapping is to use learning algorithms that only require dot products between the vectors in *V*, and choose the mapping such that these high-dimensional dot products can be computed within the original space, by means of a *kernel function* (Figure 4). Therefore, we applied the kernel types of *C-SCV, radial and dot* on the datasets to find the best accuracy.

**Figure 5:**
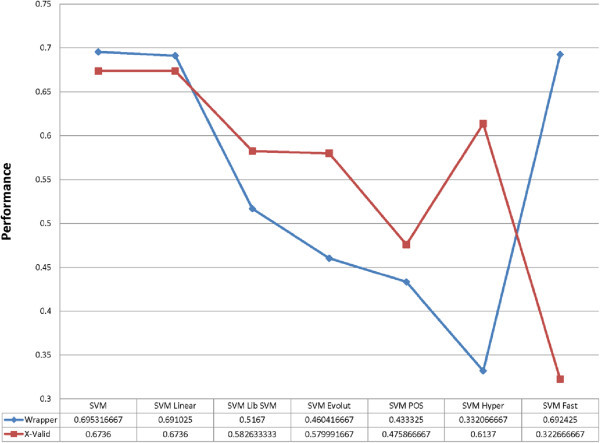
**Average performances of two validation methods (X-Validation and Wrapper-Validation) applied on seven different** ***SVM*** **algorithms (** ***SVM*** **,** ***SVM*** **Linear,** ***SVM*** **Lib,** ***SVM*** **Evolutionary,** ***SVM*** **POS,** ***SVM*** **Hyper and** ***SVM*** **Fast).**

#### Artificial neural network (ANN)

Three models of artificial neural networks algorithms ran on 13 datasets (*FCdb* and 12 datasets that obtained from attribute weighting models). The models were *Auto MLp (* multilayer perceptron), *Neural Net* and *Perceptron* (Single-layer Neural Networks). The term of "Perceptrons" was coined by Frank Rosen Blatt in 1962 and is used to describe the connection of simple neurons into networks. In computational geometry, the Single-layer Neural Networks (Perceptrons) is an algorithm for supervised classification of an input into one of two possible outputs. It is a type of linear classifier, i.e. a classification algorithm that makes its predictions based on a linear predictor function combining a set of weights with the feature vector describing a given input. For the moment we will concentrate on Single Layer Perceptrons. A *multilayer perceptron (MLP)* is a feed forward artificial neural network model that maps sets of input data onto a set of appropriate output. An MLP consists of multiple layers of nodes in a directed graph, with each layer fully connected to the next one. Except for the input nodes, each node is a neuron (or processing element) with a nonlinear activation function. MLP utilizes a supervised learning technique called back propagation for training the network (Rosenblatt 1961). MLP is a modification of the standard linear perceptron and can distinguish data that is not linearly separable (Cybenko 1989). The accuracy and Kappa values from running these three *ANN* models on 13 datasets illustrated in Table 4.

#### Naïve Bayes

A *Naïve Bayes* classifier is a simple probabilistic classifier based on applying *Bayes'* theorem with strong (naïve) independence assumptions. A more descriptive term for the underlying probability model would be "independent feature model". In simple terms, a *Naïve Bayes* classifier assumes that the presence (or absence) of a particular feature of a class is unrelated to the presence (or absence) of any other feature, given the class variable. This classifier has been widely used before (for more details see (West 2003 Baseri et al. 2011). Two models of *Naïve Bayse* (returns classification model using estimated normal distributions) and *Naïve bayse kernel* (returns classification model using estimated kernel densities) (Beiki et al. 2012) used and the model accuracy in predicting the type of lung tumor calculted.

## Electronic supplementary material

Additional file 1: Table S1: The list of protein attributes that calculated by PROFEAT server. (DOC 72 KB)
